# Supplementary material for: The interactome of intact mitochondria by cross-linking mass spectrometry provides evidence for coexisting respiratory supercomplexes
Source: Mol Cell Proteomics. 2017 Dec 8;17(2):216–32. doi: 10.1074/mcp.RA117.000470 (PMC5795388; doi:10.1074/mcp.RA117.000470)
Supplement: Supplemental Data [file supp_RA117.000470_133922_0_supp_23978_fzffwf.zip › spectra_annotation/mito_DR_spectra_annotation/386-1-2-1-3-1.pdf]

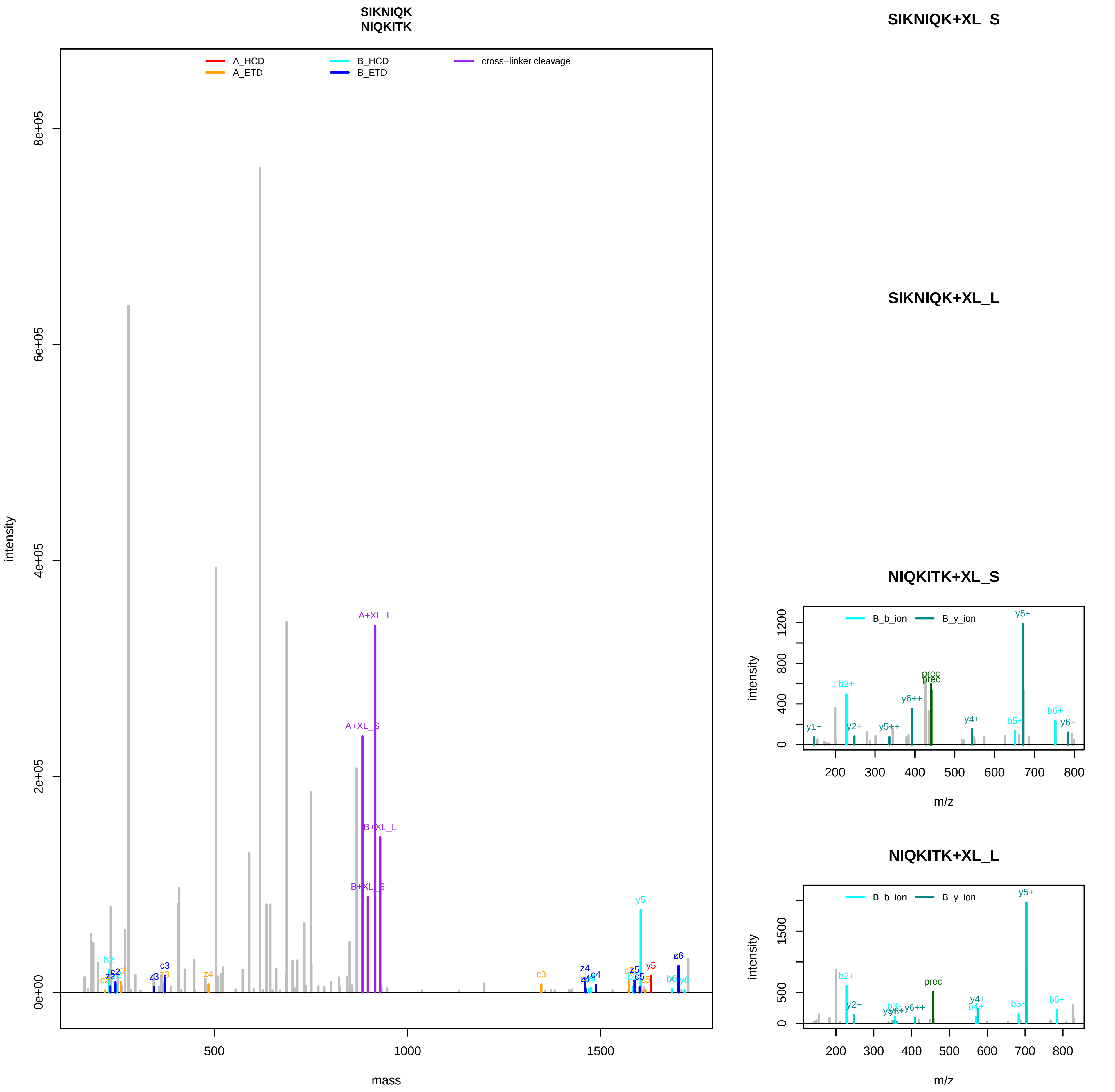

SIKNIQK
NIQKITK
A_HCD
A_ETD
B_HCD
B_ETD
cross−linker cleavage
intensity
mass
8e+05
6e+05
4e+05
2e+05
0e+00
500
1000
1500
A+XL_L
A+XL_S
B+XL_L
B+XL_S
y5
z6
b6
y6
z5
c5
y5
z4
c4
c3
z4
z3
c3
b2
z2
c2
SIKNIQK+XL_S
SIKNIQK+XL_L
NIQKITK+XL_S
B_b_ion
B_y_ion
y5+
prec
b2+
y6++
y1+
y2+
y5++
y4+
b5+
b6+
y6+
m/z
NIQKITK+XL_L
b2+
y2+
b3+
y6++
y4+
b4+
b5+
b6+
